# Comparing Internal and Interparticle Space Effects of Metal–Organic Frameworks on Polysulfide Migration in Lithium–Sulfur Batteries

**DOI:** 10.3390/nano11102689

**Published:** 2021-10-12

**Authors:** UnJin Ryu, Won Ho Choi, Panpan Dong, Jeeyoung Shin, Min-Kyu Song, Kyung Min Choi

**Affiliations:** 1 Industry Collaboration Center, Sookmyung Women’s University, Cheongpa-ro 47-gil 100, Yongsan-gu, Seoul 04310, Korea; unjin@sookmyung.ac.kr; 2 Institute of Advanced Materials and Systems, Sookmyung Women’s University, Cheongpa-ro 47-gil 100, Yongsan-gu, Seoul 04310, Korea; wonhochoi@sookmyung.ac.kr; 3 School of Mechanical and Materials Engineering, Washington State University, Pullman, WA 99164, USA; panpan.dong@wsu.edu; 4 Department of Mechanical Systems Engineering, Sookmyung Women’s University, Cheongpa-ro 47-gil 100, Yongsan-gu, Seoul 04310, Korea; 5 Department of Chemical and Biological Engineering, Sookmyung Women’s University, Cheongpa-ro 47-gil 100, Yongsan-gu, Seoul 04310, Korea

**Keywords:** lithium–sulfur battery, metal–organic frameworks, separator coating, internal space, interparticle space

## Abstract

One of the critical issues hindering the commercialization of lithium–sulfur (Li–S) batteries is the dissolution and migration of soluble polysulfides in electrolyte, which is called the ‘shuttle effect’. To address this issue, previous studies have focused on separators featuring specific chemical affinities or physical confinement by porous coating materials. However, there have been no studies on the complex effects of the simultaneous presence of the internal and interparticle spaces of porous materials in Li–S batteries. In this report, the stable Zr-based metal–organic frameworks (MOFs), UiO-66, have been used as a separator coating material to provide interparticle space via size-controlled MOF particles and thermodynamic internal space via amine functionality. The abundant interparticle space promoted mass transport, resulting in enhanced cycling performance. However, when amine functionalized UiO-66 was employed as the separator coating material, the initial specific capacity and capacity retention of Li–S batteries were superior to those materials based on the interparticle effect. Therefore, it is concluded that the thermodynamic interaction inside internal space is more important for preventing polysulfide migration than spatial condensation of the interparticle space.

## 1. Introduction

Upcoming electrochemical energy storage systems demand higher energy density, good stability, and low-cost for implementation in advanced portable electronic devices and electric vehicles. To go beyond conventional lithium-ion batteries. Lithium–sulfur (Li–S) batteries have received much attention as one of the most promising candidates for a next-generation secondary battery systems because of their high theoretical energy density (2600 Wh kg^−1^) and cost-effectiveness due to the abundance of elemental sulfur on earth [1,2]. However, there are major issues that hinder the practical application of Li–S batteries, notable among which is the ‘shuttle effect’ caused by the dissolution and diffusion of highly soluble polysulfide intermediates in electrolytes during the discharge/charge processes. Polysulfide dissolution leads to active material loss from the sulfur cathode, resulting in fast capacity decay and poor cycle life [3]. To mitigate the shuttle effect, many efforts have been devoted to blocking soluble polysulfide intermediates via physical and chemical confinement of separators by introducing various coating materials. [3,4,5,6,7,8]. As for physical confinement, porous materials such as porous carbon, graphene, and metal oxide were used to prevent the penetration of polysulfides from cathode to anode [5,9,10,11]. As for chemical confinement, the functional groups of the separator coating materials are optimized to chemically attract polysulfides in the cathode side [5,8,12,13]. However, these materials with the physical and chemical confinement possess interparticle void spaces in the separators. The effects of these interparticle spaces on the extent of polysulfide migration in Li–S batteries are yet to be studied.

Metal–organic frameworks (MOFs) have been applied as the separator coating materials and host materials owing to their distinct advantages, such as, a well-ordered porous matrix, high surface area, tunable morphology and pore structure, and amenability to functionalization [14,15,16,17,18,19,20,21,22,23,24,25]. Therefore, the chemical and physical properties of MOF structures can be easily adjusted as necessary. Their particle sizes can also be conveniently controlled [26]. Recently, there have been various attempts to confine polysulfides via physical confinement using separators coated and filtrated with MOFs. Four different MOFs, Y-FTZB, ZIF-7, ZIF-8, and HKUST-1, were used as coating materials for separators to enhance the mitigation of polysulfide diffusion with various pore structures and particle morphologies [27]. In consideration of the chemical effect, studies have been conducted in which MOFs are modified with specific functional groups [28], and defect sites [29] have been applied to separators. Despite the controllable internal space of MOFs, the interparticle spaces still exist when they are applied as separators. As the polysulfide intermediates can migrate through interparticle spaces, the ability of these interparticle spaces to block polysulfide migration in Li–S batteries warrants further investigation. Moreover, a comparison of the efficiency with internal space control techniques involving chemical and physical moieties is necessary.

**Figure 1:**
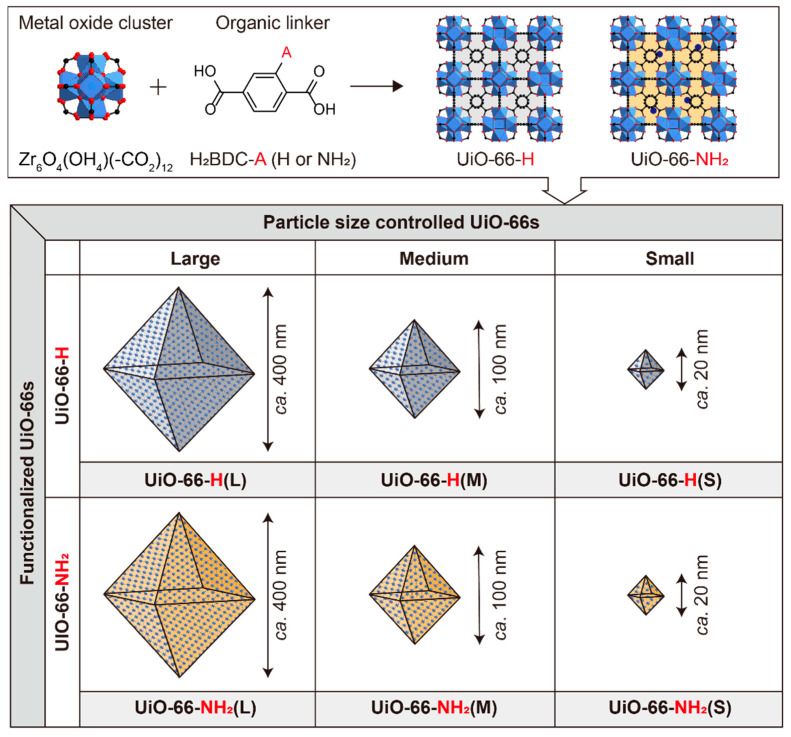
Schematic diagram for the synthesis of UiO-66-H(X) and UiO-66-NH_2_(X) in three different sizes: large (L), medium (M), and small (S).

In this study, the effects of interparticle space have been compared with those of the internal spaces of MOFs in MOF-coated separators. Specially designed MOFs, featuring controlled particle sizes and functional groups, were used as a coating for separators in Li–S batteries, and their cycling performances were compared to determine the effect of their interparticle and internal spaces on polysulfide migration in Li–S batteries. UiO-66 was used as a representative MOF because of its high physical and chemical stability. The UiO-66s were functionalized with H and NH_2_ (UiO-66-H and UiO-66-NH_2_, respectively) for internal space control. The particle sizes of these MOFs were controlled to be large (L, ca. 400 nm), medium (M, ca. 100 nm), and small (S, ca. 20 nm) for meticulous interparticle space control. These MOFs are referred to as UiO-66-H(X) and UiO-66-NH_2_(X) where X indicates the size of the MOF (Figure 1). After thorough characterizations, including the identification of particle size, morphology, and functionalities, the polysulfide absorption performances of these samples were evaluated. All these MOFs were coated on polypropylene separators with a polyvinylidene fluoride (PVDF) binder (9:1, w/w) using the doctor blade method. The MOF particles were found to exhibit distinct void tendencies and thus, in the case of larger MOFs, broader interparticle spaces, that is, voids were available for the migration of polysulfide intermediates in the MOF coated separators. All the UiO-66 separators were applied to Li–S batteries, and their performances were compared using galvanostatic charge/discharge profiles and electrochemical impedance spectroscopy (EIS). Their long-term cycling performances were also studied. Li–S batteries using the UiO-66-NH_2_(X) series consistently exhibited higher specific capacity than those using the UiO-66-H(X) series, which is attributed to the strong adsorption of amine groups in UiO-66-NH_2_(X) towards soluble polysulfides. Consequently, the internal space effect of MOFs was found to be much stronger than their interparticle space effects towards the blocking of polysulfide migration in electrolytes.

Previous studies using MOFs as separator coating materials have mostly focused on improving the cycling stability of batteries by introducing functional groups in the internal spaces [28,29]. Although there are other studies involving morphology control of MOFs [19,30,31], the comparison between the effect of internal and interparticle spaces of MOFs on the performances of Li–S batteries is yet to be reported. This study demonstrates the advantages of the internal thermodynamic attraction of MOFs in comparison to the interparticle space effects by controlling their particle sizes, which will provide a new direction to design functional separators for improving Li–S battery performance.

## 2. Materials and Methods

### 2.1. Materials

Terephthalic acid (98%), 2-aminoterephthalic acid (99%), triethylamine (TEA, ≥99.5%), *N,N*-dimethylformamide (DMF, ≥99.8%), 1,3-dioxolane (DOL, anhydrous, 99.8%), 1,2-dimethoxyethane (DME, anhydrous, 99.5%), lithium sulfide (99.98%), polyvinylidene Fluoride (PVDF) (MTI corporation), super-P 45 carbon (IMERYS), *N*-methyl-2-pyrrolidone (NMP), and bis(trifluoromethane)sulfonimide lithium salt (LiTFSI, 99.95%) were purchased from Sigma-Aldrich Chemicals, Burlington, MA, USA. Zirconium (IV) chloride (ZrCl_4_, 99.5+%), acetic acid (glacial, 99.9+%), and sulfur powder (sublimed, 100 mesh, 99.5%) were purchased from Alfa Aesar, Tewksbury, MA, USA. Carbon nanotubes (CNTs, –COOH functionalized multiwalled, 95+%) were purchased from Nanostructured & Amorphous Materials Inc, Los Alamos, NM, USA.

### 2.2. Synthesis of UiO-66-H and UiO-66-NH_2_

Ligand solutions were prepared by dissolving terephthalic acid (H_2_BDC, 0.15 mmol) (for UiO-66-H) or 2-aminoterephthalic acid (H_2_BDC-NH_2_, 0.15 mmol) (for UiO-66-NH_2_) in DMF (5 mL). Separately, ZrCl_4_ (0.15 mmol) was dissolved in DMF (5 mL) and acetic acid (0.69 mL). This solution served as the metal source. The ligand and metal salt solutions were combined in a 20 mL glass vial. For synthesizing three distinctly sized UiO-66s, different quantities of TEA were added to the solutions as follows: 0 μL (for large size), 30 μL (for medium size), and 60 μL (for small size). The vial was placed directly in an aluminum heating block at 120 °C for 16 h. After the reaction, the vial was removed and cooled to room temperature. The powder product was separated using a centrifuge (6000 rpm for 10 min) followed by washing with DMF and methanol. After washing, the product was dried in a vacuum oven.

### 2.3. Fabrication of UiO-66-H (or UiO-66-NH_2_) Separators

The synthesized UiO-66-H (or UiO-66-NH_2_) nanoparticles were mixed with PVDF binder in NMP at a mass ratio of 9:1 (UiO-66s:PVDF). The mixture was ground via ball-milling to obtain a homogeneous slurry. The slurry was coated onto Celgard 2400 (polypropylene membrane, PP) using the doctor blade method. The coated separator was dried overnight in a vacuum oven at 60 °C. The obtained UiO-66s separator had approximately 20 μm thickness of MOF layer and was cut into a disk of diameter 16 mm.

### 2.4. Preparation of S/CNT Cathode and Battery Assembly

Sulfur was ground with carbon nanotubes (CNTs) at a mass ratio of 7:3 to a homogeneous colored powder. Then, the powder mixture was heated from room temperature to 155 °C (holding for 12 h) and then through 260 °C (holding for 0.5 h) in a tube furnace under an argon atmosphere to enable the infiltration of sulfur into CNTs. The S/CNT composites were mixed with carbon black (Super-P 45 and PVDF binder in NMP at a mass ratio of 7:2:1 (S/CNT:Super-P:PVDF). The mixture was ground via ball milling and coated onto aluminum foil using the doctor blade method. Thereafter, the electrode was dried in a vacuum oven at 60 °C, punched to a diameter of 12mm and kept in an argon-filled glove box (H_2_O < 0.5ppm, O_2_ < 0.5ppm). The mass loading of sulfur in the electrode was ca. 0.6–1.0 mg/cm^2^. Coin cells (CR2032 type, Wellcos Corp., Gunpo, Korea) were assembled with lithium metal disks (15.6 mm diameter) as anodes and S/CNT as cathodes. The electrolyte was composed of 1 M LiTFSI dissolved in DOL/DME mixed solution (1:1, *v*/*v*) with 0.2 M LiNO_3_. The configuration of the assembled cell is represented as S/CNT cathode (12 mm)/UiO-66s coated separator (16 mm)/electrolyte (17 μL)/bare PP separator (18 mm)/electrolyte (17 μL)/Li metal anode (15.6 mm). The UiO-66s-coated layer was facing to the cathode side.

### 2.5. Material Characterizations

Powder X-ray diffraction (PXRD) spectra were obtained using a Bruker instrument (D8 Advance) at 1600 W (40 kV, 40 mA). The scanning speed was 0.4 s/step at 0.04° increments. Nitrogen adsorption analysis was performed on a BELSORP-max automatic volumetric gas adsorption analyzer and the sample was activated by evacuating at 120 °C for 24 h. The morphology of MOF particles and coated separators was evaluated using a field emission scanning electron microscope (FE-SEM, JEM-7600F, JEOL, Tokyo, Japan). Attenuated total reflection Fourier transform infrared (ATR-FTIR) spectroscopy was performed using a Nicolet iS50 FTIR spectrometer (Thermo Scientific, Waltham, MA, USA). The UV-visible absorption spectra were recorded using a Shimadzu UV-2600 instrument with quartz cuvettes.

### 2.6. Electrochemical Analysis

The galvanostatic charge/discharge measurements were performed using a BTS4008 battery cycler (NEWARE, Hong Kong, China) between 1.8 to 2.8 V (vs. Li/Li^+^) under the current densities of 100 mA/g and 250 mA/g. All specific capacities in this work were calculated based on the mass of sulfur. EIS experiments were performed in a frequency range from 1 MHz to 10 mHz using a potentiostat (SP-200, BioLogic, Seyssinet-Pariset, France) with an AC oscillation amplitude of 10 mV.

## 3. Results and Discussion

Zirconium-based MOFs are both chemically and electrochemically stable in battery systems [32,33]. In this work, UiO-66 was chosen as the separator coating materials because its structure is devoid of unsaturated metal sites that can interact with reactive ions in organic electrolyte. UiO-66 is a three-dimensional structure composed of benzene ring-based terephthalate ligands and two types (tetrahedral cage (9 Å) and octahedral cage (11 Å)) of regularly arranged intrinsic micropores with a 0.6 nm triangular window [22]. The pore sizes of UiO-66 were favorable for trapping and blocking soluble polysulfides (Li_2_S_n_, 4 < n ≤ 8). In addition, the chemical environments of the pores can be conveniently modified by replacing the H in terephthalate with targeted functional groups such as −NH_2_, −OH, and −COOH.

### 3.1. Preparation of UiO-66s Nanoparticles

The UiO-66s were prepared via a typical solvothermal synthesis that was modified from a reported method [34] to control their particle size and functionality to enable the comparison of interparticle and internal space effects. Terephthalic acid (H_2_BDC), ZrCl_4_, and acetic acid were dissolved in 20 mL DMF in a Teflon-lined vial. To this solution, different doses of TEA were added to obtain UiO-66s of varying particle sizes. TEA was used to deprotonate the carboxyl groups of organic linkers and ensure dominant nucleation to produce UiO-66s of small particle sizes. The larger amount of TEA used, the smaller the UiO-66 particle size. This mixture was sealed and placed at 120 °C for 16 h in an aluminum heating block. The heating block has a high heat transfer rate, which is advantageous for synthesizing uniform nano-sized MOFs. The white suspensions were collected and washed with DMF and methanol using a centrifuge. The obtained MOFs were then immersed in methanol for three repeated 24 h periods to activate their pores. The UiO-66-H(X) particles were separated and dried under vacuum overnight. The same synthetic procedure was followed to obtain UiO-66-NH_2_(X) particles except that the ligand reagent was changed to 2-aminoterephthalic acid. All products were characterized using PXRD, SEM, FT-IR, and N_2_ adsorption isotherms measurements to determine the intrinsic crystallinity, morphology, functionality, and permanent porosity of UiO-66s particles, respectively, which will be discussed in the following sections.

### 3.2. Physicochemical Characterizations of UiO-66s Nanoparticles

**Figure 2:**
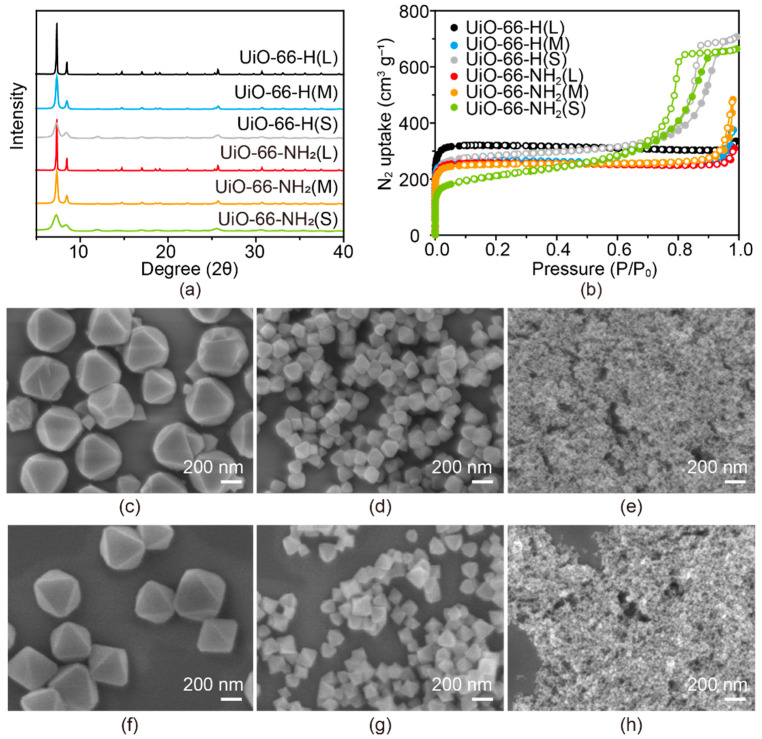
Characterizations of UiO-66-H(X) and UiO-66-NH_2_(X): (**a**) PXRD patterns; (**b**) N_2_ adsorption-desorption isotherms. SEM images of (**c**) UiO-66-H(L), (**d**) UiO-66-H(M), (**e**) UiO-66-H(S), (**f**) UiO-66-NH_2_(L), (**g**) UiO-66-NH_2_(M), and (**h**) UiO-66-NH_2_(S).

The PXRD patterns of both UiO-66-H(X) and UiO-66-NH_2_(X) samples matched well with those simulated results from their crystal structures, indicating that they possess the same UiO-66 crystal structure (Figure 2a and Figure S1). As the particle size increased, sharper diffraction peaks appeared, indicating higher crystallinity. The permanent porosity was confirmed by N_2_ adsorption measurements as shown in Figure 2b and Table S1. The adsorption behaviors of all UiO-66-H(X) and UiO-66-NH_2_(X) species were similar in micropore filling regions at low relative pressure. However, both the UiO-66-H(S) and UiO-66-NH_2_(S) showed hysteresis at high relative pressures (P/P_0_) above 0.6, which was different from the behaviors of UiO-66-H(L) and UiO-66-NH_2_(L). This is because UiO-66-H(S) and UiO-66-NH_2_(S) had small interparticle spaces corresponding to those of mesoscale pores (Figure 2b). In contrast, UiO-66-H(L) and UiO-66-NH_2_(L) had large interparticle spaces that were beyond the scope of N_2_ adsorption measurements. SEM images depict the high uniformity of UiO-66-H(X) and UiO-66-NH_2_(X) structures along with octahedral geometry that is typical of UiO-66. As shown in Figure 2c–h, both UiO-66-H(X) and UiO66-NH_2_(X) samples have large (L), medium (M), and small (S) particle sizes of ca. 400 nm, 100 nm, and 20 nm, respectively. Consistent with previous PXRD results, UiO-66-H(X) and UiO-66-NH_2_(X) samples show sharp edges in their octahedral shapes indicating high crystallinity. These results confirmed that the prepared crystals of UiO-66-H(X) and UiO-66-NH_2_(X) had the desired particle sizes and interparticle spaces.

### 3.3. Li_2_S_6_ Absorption Measurements

**Figure 3:**
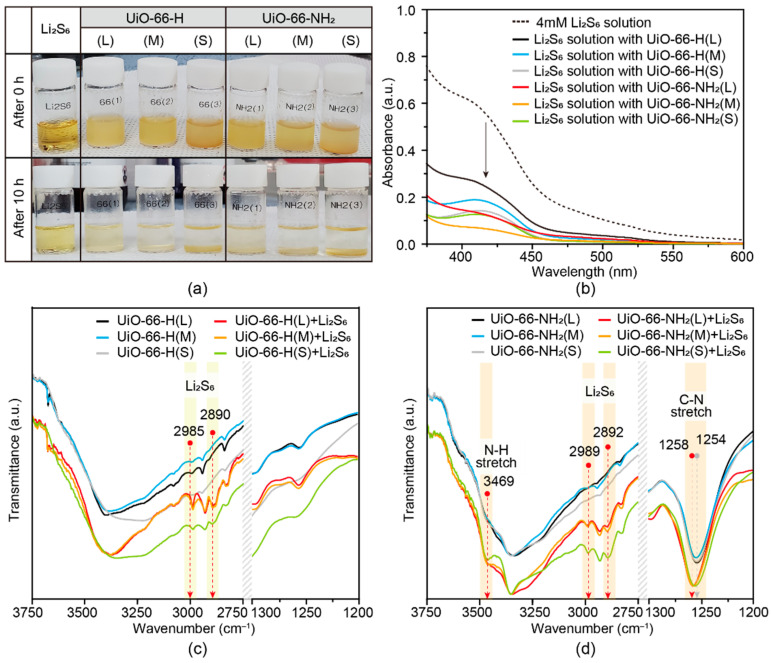
Li_2_S_6_ absorption analysis of UiO-66-H(X) and UiO-66-NH_2_(X) powders: (**a**) Digital image of 4 mM Li_2_S_6_ solution before and after soaking UiO-66-H(X) and UiO-66-NH_2_(X) powders for visual absorbing analysis; (**b**) UV-visible spectra of Li_2_S_6_ solution before and after 10 h treatment with UiO-66-H(X) and UiO-66-NH_2_(X). FT-IR spectra of: (**c**) UiO-66-H(X) and (**d**) UiO-66-NH_2_(X) before and after absorbing Li_2_S_6_.

As polysulfides (Li_2_S_n_, 4 < n ≤ 8) are soluble in electrolyte, it is important to block them in the cathode side by the coating materials on the separator to further mitigate the shuttle effect in Li–S batteries. Absorption experiments of polysulfides were performed by immersing UiO-66-H(X) and UiO-66-NH_2_(X) samples in Li_2_S_6_ (DOL/DME, 1:1, *v*/*v*) solution. The Li_2_S_6_ solution was prepared according to a previously reported method [35]. The yellow color of Li_2_S_6_ solution faded instantly and became colorless. It was kept on observation for 10 h, while the UiO-66 powder turned from a white to yellow color (Figure 3a and Figure S2), suggesting that polysulfide molecules were absorbed by the pores of UiO-66s from the DOL/DME solvent. The supernatants were then analyzed using UV-visible spectroscopy. The UV-visible spectra were compared to evaluate the relative absorption capacities of various UiO-66s samples towards polysulfides after 10 h. As shown in Figure 3b, the original Li_2_S_6_ stock solution had an absorbance of approximately 0.6 at 320 nm. Hence, absorbance values lower than 0.6 originated from the absorption of polysulfides in UiO-66 samples. In the case of UiO-66-H(X) samples, the absorption ability was obviously affected by particle size: the absorbance at 320 nm decreased gradually with a decrease in particle size. On the other hand, as for the UiO-66-NH_2_(X) species, no significant differences in absorbance were observed with different particle sizes. Moreover, the absorbance of UiO-66-NH_2_(X) samples was much lower than that of UiO-66-H(X) samples. These results indicate that the amine functionality in UiO-66-NH_2_(X) imparts a strong affinity towards polysulfides, overwhelming the differences in their particle sizes.

To examine the interaction between UiO-66s and polysulfides, FT-IR analysis of all UiO-66s particles collected from the Li_2_S_6_ solution via centrifugation was carried out. The FT-IR spectra were measured via ATR analysis by placing the dried powder samples on a diamond crystal plate. In addition, to measure the FT-IR spectra of the Li_2_S_6_ solution, the sample was prepared by dropping liquid directly on a crystal plate followed by drying. Before adding UiO-66s to the Li_2_S_6_ solution, UiO-66-H(X) samples showed identical FT-IR patterns regardless of their particle sizes. As shown in Figure 3c, after Li_2_S_6_ immersion treatment, two new peaks appeared at 2985 and 2890 cm^−1^ for all UiO-66-H(X) samples, suggesting the absorption of Li_2_S_6_ (Figure S3). Absorption of soluble polysulfide by the micropores of UiO-66-H(X) was thus confirmed. In a similar manner, the UiO-66-NH_2_(X) species showed identical FT-IR patterns regardless of their particle sizes and the presence of Li_2_S_6_ peaks appeared at 2989 and 2892 cm^−1^ (Figure 3d), indicating the absorption of Li_2_S_6_. However, in the case of UiO-66-NH_2_(X) samples, the peak corresponding to C–N stretching at 1254 cm^−1^ was shifted to 1258 cm^−1^. Additionally, the intensity of the peak at 3469 cm^−1^ that was assigned to N–H stretching increased after Li_2_S_6_ absorption treatment (Figure 3d and Figure S4). It is attributed to the hydrogen bonding interaction between Li_2_S_6_ and amine functional groups [36] in the UiO-66-NH_2_ pores.

In conjunction with the UV-vis and FT-IR results, UiO-66-H(X) and UiO-66-NH_2_(X) samples could absorb polysulfides by their micropores like a molecular sieve. While the particle size has a distinct influence on the polysulfides absorption ability of UiO-66s, the internal space interactions derived from amine groups had much stronger effects. Therefore, the UiO-66-NH_2_(X) samples exhibited much more effective polysulfide absorption than UiO-66-H(X) sampled in all size ranges.

### 3.4. Morphological Characterizations of MOF-Coated Separators

**Figure 4:**
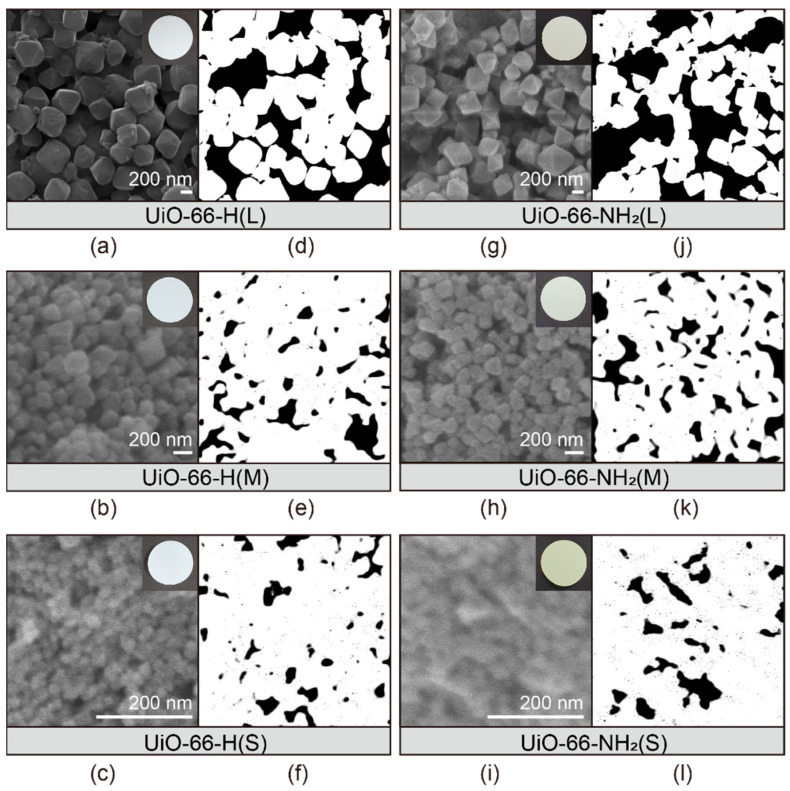
Morphology of MOF-coated separators and their interparticle spaces. SEM images depicting morphology of MOF-coated separator surfaces for: (**a**–**c**) UiO-66-H(X) and (**g**–**i**) UiO-66-NH_2_(X) samples. The images after threshold adjustment with: (**d**–**f**) UiO-66-H(X) and (**j**–**l**) UiO-66-NH_2_(X) coated separators.

The UiO-66s-coated separators were fabricated by a solution casting method. Each MOF sample was mixed with a PVDF binder and an NMP to obtain a homogenous slurry that was then coated on a PP separator using the doctor blade method. All MOF-coated layers had a uniform thickness of 20 μm on the PP membrane. As shown from the digital photos (insets of Figure 4), the UiO-66-H(X) and UiO-66-NH_2_(X) coated separators (diameter: 16 mm) exhibited a uniform distribution of MOF particles. The interparticle spaces for each separator are represented by the black regions in the contrast images (Figure 4d–f,j–l) which are converted from the SEM images in Figure 4a–c,g–i, respectively. The particle sizes and percentages of internal/interparticle spaces at the surface of all MOF-coated separators are summarized in Table 1. For similar particle size ranges, the UiO-66-H(X) coated separators showed a smaller void space compared with the UiO-66-NH_2_(X) ones. Moreover, as the void space decreased, the coated layer became dense when the particle size was small, which is consistent with the N_2_ adsorption analysis of UiO-66-H(X) and UiO-66-NH_2_(X) (Figure 2b). It was confirmed that the size of MOF particles drove distinct void tendencies and the smaller MOFs lead to the narrower interparticle spaces, thereby blocking the polysulfides migration through the MOF coated separators.

### 3.5. Electrochemical Performance of Li–S Batteries with MOF-Coated Separators

**Figure 5:**
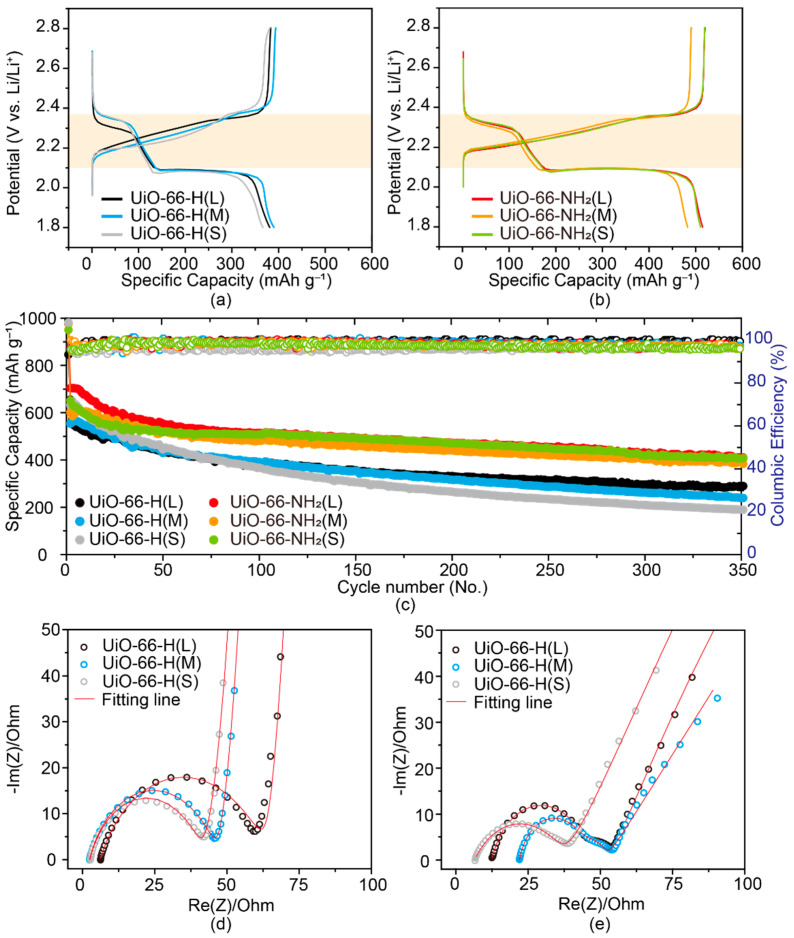
Electrochemical performance of Li–S batteries with MOF-coated separators at 250 mAg^−1^. Voltage profiles at 250 mAg^−1^ (the 100th cycle) for cells assembled with: (**a**) UiO-66-H(X) and (**b**) UiO-66-NH_2_(X) coated separators. (**c**) Long-term cycling performance of UiO-66-H(X) and UiO-66-NH_2_(X) with first cycle at 100 mAg^−1^. Nyquist plots of (**d**) fresh cells and (**e**) after 350 cycles using UiO-66-H(X) coated separators.

The fabricated MOF-coated separators were employed in Li–S batteries to investigate the internal and interparticle space effects on the polysulfide migration. The CR2032 type coin cells were assembled with S/CNT as the cathode (CNTs used as sulfur-hosting materials, MOF-coated separators (UiO-66-H(X) and UiO-66-NH_2_(X)) coated on PP membrane), a lithium metal disk as the anode and 1 M LiTFSI-DOL/DME (1:1, *v*/*v*) with 0.2 M LiNO_3_ as the electrolyte. All cells were tested under the current density of 250 mA g^−1^ in the voltage range of 1.8–2.8 V (vs. Li/Li^+^) after the activation step at 100 mA g^−1^ for the first cycle (Figure S6). As shown in Figure 5a,b, the galvanostatic charge/discharge profiles showed two typical voltage plateaus in both UiO-66-H(X) and UiO-66-NH_2_(X) cells. During discharging, elemental sulfur was reduced to soluble Li_2_S_n_ (4 < n ≤ 8) and finally insoluble Li_2_S_2_/Li_2_S with two main voltage plateaus at 2.3 V and 2.1 V (vs. Li/Li^+^) [37]; during charging, the insoluble Li_2_S_2_/Li_2_S were oxidized back to S_8_ via the soluble polysulfides intermediates. These behaviors showed that both UiO-66-H(X) and UiO-66-NH_2_(X) had no electrochemical influence on the typical charge/discharge redox process in the Li–S battery. Nevertheless, cells with UiO-66-NH_2_(X) separators exhibited higher specific capacities than those with UiO-66-H(X) ones, indicating the enhanced utilization of sulfur because of the strong adsorption ability of amine groups towards polysulfides as discussed from UV-vis results in Figure 3b.

Although there was no noticeable polarization difference among the UiO-66-H(X) species, significant differences were observed in the long-term cycling performances (Figure 5c). UiO-66-H(L) exhibited a higher capacity retention of 49.7% (initial: 546.15 mAh g^−1^ and final: 271.20 mAh g^−1^) than UiO-66-H(M) and UiO-66-H(S) with capacity retention values of 42.2% (initial: 567.19 mAh g^−1^ and final: 239.48 mAh g^−1^) and 29.2% (initial: 649.4 mAh g^−1^ and final: 189.76 mAh g^−1^), respectively, for the 350th cycle. The cross point of capacity fading appeared after 75 cycles depending on the particle size. Smaller particles are coated on the PP separators less reliably than larger particles having larger interparticle spaces. We also summarized the previously reported UiO-66 coated separators in Li–S batteries in Table S2. These reports show the enhanced cycling performance of Li–S batteries compared with the batteries using pristine separators, which confirms the effective mitigation of polysulfides by UiO-66 or UiO-66-NH_2_. In this work, we further studied the effects of internal and interparticle space on polysulfide migration via size control and amine group modification, which could fill the gap between the microstructure of UiO-66s and their electrochemical application. To understand the effect of particle size on the cyclability of UiO-66-H(X) cells, EIS were conducted for investigating the reaction governing the conversion of soluble polysulfide to insoluble Li_2_S over three different frequency regions: high-frequency region, middle-frequency region, and low-frequency region. In the high-frequency region (typically over 10 kHz), the series ohmic resistances (R_s_) of the electrolyte, soluble polysulfide, and electrodes dominate over their diffusion. In the middle-frequency region, charge transfer resistance (R_ct_) reveals electronic resistance at the solid-liquid interfaces. In the low-frequency region, Warburg impedance (Z_w_) is observed, that is, diffusion dominates over the ohmic resistance. Therefore, each frequency region provides insight into the properties of the resistance and the diffusion as a function of the degree of interparticle spaces. In the Nyquist plot (Figure 5d), R_s_, R_ct_, and Z_w_ were estimated by EIS fitting using an equivalent circuit model (Figure S7). From the EIS fitting results, R_s_ and R_ct_ of UiO-66-H(L), UiO-66-H(M), and UiO-66-H(S) cells were estimated to be 6.14 Ω and 56.3 Ω, 2.474 Ω and 43.21 Ω, and 2.585 Ω and 38.06 Ω, respectively. The R_s_ and R_ct_ values of UiO-66-H(S) are smaller than those of UiO-66-H(M) and UiO-66-H(L) (Table S3), indicating that the presence of interparticle spaces increases the electric resistance and ionic conductivity, as reported in previous work [38]. Accordingly, smaller particle sizes caused the enhanced electric and ionic conductivity by reducing the kinetic barrier of the redox reactions [39], resulting in a high initial capacity which is reflected in the enhanced R_s_ and R_ct_ of UiO-66-H(S).

On the other hand, Z_w_ values exhibit the opposite tendency of becoming reduced with increasing interparticle space fractions. In the low-frequency region, Z_w_ values were strongly related to diffusion via MOF particles within the separator, indicating that the diffusion of soluble polysulfide can be regulated by controlling the balance between internal and interparticle spaces. The Z_w_ values of UiO-66-H(L), UiO-66-H(M), and UiO-66-H(S) are 14.76 Ω s^−1/2^, 16.28 Ω s^−1/2^, and 21.35 Ω s^−1/2^, respectively. Smaller Z_w_ values signify faster diffusion of ions, such as polysulfide and lithium ion. Thus, these results imply that diffusion depends on the interparticle spaces, although UiO-66-H(S) exhibited better adsorption of Li_2_S_6_ than UiO-66-H(M) and UiO-66-H(L). The Nyquist plots of the 350th cycles were fitted to reaffirm the diffusion tendency of UiO-66-H(L) using a modified equivalent circuit model (Figure S8). After 350 cycles, the Z_w_ value of UiO-66-H(L) was 6.906 Ω s^−1/2^, which is much smaller than that of UiO-66-H(M) (9.265 Ω s^−1/2^) and UiO-66-H(S) (29.93 Ω s^−1/2^) (Table S4). The Z_w_ values of UiO-66-H(L) and UiO-66-H(M) decreased after 350 cycles, implying that the diffusion was accelerated by the interparticle spaces after repeated charging and discharging. As for UiO-66-H(S) cells, the Z_w_ value increased conspicuously after 350 cycles, suggesting that the insufficient interparticle spaces hindered diffusion during cycling because of restricted mass transport channels. Therefore, densely packed particles did not effectively prevent the shuttle effect of polysulfides due to the limited diffusion into the micropores of UiO-66-H(X). The passing soluble Li_2_S_n_ (4 < n ≤ 8) resulted in the irreversible formation of insoluble Li_2_S_2_/Li_2_S on lithium anode as shown in the capacity degradation. On the contrary, large interparticle spaces of UiO-66-H(L) promoted ion diffusion despite an increase in electric resistance, leading to good capacity retention after the 350th cycle.

Subsequently, the internal space effects in UiO-66-NH_2_(X) and UiO-66-H(X) were compared. As demonstrated in Figure 5c, the initial capacity and capacity retention of UiO-66-NH_2_(X) cells are much higher than those of UiO-66-H(X) ones. Moreover, there is no significant capacity difference among the UiO-66-NH_2_(X) species. This was because the amine functional groups had a significantly larger preventive influence on the shuttle effect of soluble polysulfides than the interparticle space effects. In the initial reduction step of elemental sulfur, soluble polysulfide intermediates were trapped on the coated separators. The EIS fitting parameter of UiO-66-H(X) demonstrated that the existence of sufficient interparticle spaces improved the diffusion of polysulfide intermediates. Additionally, the amine group strengthened the Van der Waals force between the trapped polysulfides and UiO-66-NH_2_(X) via the unpaired-electrons of nitrogen, thus making them more polar. According to the literature on nitrogen effect, the incorporation of nitrogen enhanced ion mobility by providing a thermodynamically favored route for diffusion [40]. To verify the role of amine, the absorption test of UiO-66s coated separators towards Li_2_S_6_ was carried out (Figure S9). The color of Li_2_S_6_ solutions containing UiO-66-NH_2_(X) coated separators especially UiO-66-NH_2_(L) are brighter than those containing UiO-66-H(X) ones. The internal space effect originating from the enhanced absorption of the amine group is strong enough to suppress polysulfide migration through separators regardless of the interparticle space effects, that is, the difference in void spaces. This result also proved that the effect of internal space on the chemical affinity surpassed the effect of interparticle space arising from particle size control.

## 4. Conclusions

Through this study, we discovered the key factors in preventing polysulfides migration in Li–S batteries. By using UiO-66s as coating materials in which the pore structure and framework are equally controlled, separators were designed with three different UiO-66 particle sizes (large, medium, and small) by controlling the balance between their internal and interparticle spaces. Furthermore, amine functional groups were introduced into UiO-66s to enhance the absorption ability towards polysulfides. We found that Li–S cells with UiO-66-H(x) separators exhibited distinct cycling performances, which greatly depends on the particle sizes and interparticle space of UiO-66-H(X). However, this interparticle space effect became inconspicuous when the internal space caused by amine groups was the dominated effect on the cyclability of Li–S cells. Therefore, the specific capacity and cycling performance of Li–S cells with UiO-66-NH_2_(X) separators were much improved by the amine groups regardless of the difference in interparticle spaces. While many studies have been conducted on the performance improvement of separators using MOFs with different structures, pore sizes, metal clusters, and organic ligands, in this study, we determined that the internal space effect is more critical to mitigating polysulfide migration than interparticle space effects when other factors that could potentially cause side effects were carefully controlled. We expect that the findings in this study will contribute to the optimized design and manufacturing of MOF-modified separators in other battery systems such as sodium–sulfur batteries.

## Figures and Tables

**Table 1:** Statistical data of UiO-66-H(X) and UiO-66-NH_2_(X) particles on the surface of MOF-coated separators comparing internal space and interparticle space proportions.

Sample Name	Particle Size (nm)	Internal Space (%)	Interparticle Space (%)
UiO-66-H(L)	ca. 400	61.4	38.6
UiO-66-H(M)	ca. 100	79.2	20.8
UiO-66-H(S)	ca. 20	84.0	16.0
UiO-66-NH_2_(L)	ca. 400	53.3	46.7
UiO-66-NH_2_(M)	ca. 100	73.0	27.0
UiO-66-NH_2_(S)	ca. 20	83.2	16.8

## Supplementary Materials

The following are available online at https://www.mdpi.com/article/10.3390/nano11102689/s1, Figure S1: PXRD patterns of simulated UiO-66, UiO-66-H(L), and UiO-66-NH_2_(L). Figure S2: Digital images of 4 mM Li_2_S_6_ solution before and after soaking UiO-66s powders for 10 h for visual analysis of absorption. Figure S3: FT-IR spectra of Li_2_S_6_ solution after drying on a diamond ATR plate. Figure S4: (a) FT-IR spectra of UiO-66-H(X) before and after absorbing polysulfide at 750–4000 cm^−^^1^. (b) FT-IR spectra of UiO-66-NH_2_(X) before and after absorbing polysulfide at 750–4000 cm^−^^1^. Figure S5: SEM images of the surface of MOF-coated separators made of (a) UiO-66-H(L), (b) UiO-66-H(M), (c) UiO-66-H(S), (d) UiO-66-NH_2_(L), (e) UiO-66-NH_2_(M), and (f) UiO-66-NH_2_(S). Figure S6: Voltage profiles of UiO-66-H(S),(L), and UiO-66-NH_2_(S),(L) tested under 100 mA g^−^^1^. Figure S7: Nyquist plots of fresh Li–S cells with UiO-66-H(X) coated separators. Figure S8: Nyquist plots of Li–S cells after 350 cycles with UiO-66-H(X)-coated separators. Figure S9: Digital images of Li_2_S_6_ solution before and after soaking MOF-coated separators for 24 h for visual analysis of absorption. Table S1: Pore structure parameters of UiO-66s. Table S2: The comparison of electrochemical performance of reported UiO-66 coated separators in Li–S batteries. Table S3: EIS fitting parameters of fresh Li–S cells with UiO-66-H(X) coated separators. Table S4: EIS fitting parameters of Li–S cells with UiO-66-H(X) coated separators after 350 cycles. Refs. [41,42,43,44] are cited in the supplementary materials.
