# Supplementary material for: Comparing Internal and Interparticle Space Effects of Metal–Organic Frameworks on Polysulfide Migration in Lithium–Sulfur Batteries
Source: Nanomaterials (Basel). 2021 Oct 12;11(10):2689. doi: 10.3390/nano11102689 (PMC8537144; doi:10.3390/nano11102689)
Supplement: Supplementary file 1 [file nanomaterials-11-02689-s001.zip › nanomaterials-1378647-supplementary.pdf]

nanomaterials

Supplementary Materials

Comparing Internal and Interparticle Space Effects of Metal–Organic Frameworks on Polysulfide Migration in Lithium–Sulfur Batteries

UnJin Ryu [1], Won Ho Choi [2], Panpan Dong [3], Jeeyoung Shin [2,4,*], Min-Kyu Song [3,*] and Kyung Min Choi [1,2,5,*]

[1] Industry Collaboration Center, Sookmyung Women's University, Cheongpa-ro 47-gil 100, Yongsan-gu, Seoul 04310, Korea; unjin@sookmyung.ac.kr
[2] Institute of Advanced Materials and Systems, Sookmyung Women's University, Cheongpa-ro 47-gil 100, Yongsan-gu, Seoul 04310, Korea; wonhochoi@sookmyung.ac.kr
[3] School of Mechanical and Materials Engineering, Washington State University, Pullman, WA 99164, USA; panpan.dong@wsu.edu
[4] Department of Mechanical Systems Engineering, Sookmyung Women's University, Cheongpa-ro 47-gil 100, Yongsan-gu, Seoul 04310, Korea
[5] Department of Chemical and Biological Engineering, Sookmyung Women's University, Cheongpa-ro 47-gil 100, Yongsan-gu, Seoul 04310, Korea
* Correspondence: jshin@sookmyung.ac.kr(J.S.); minkyu.song@wsu.edu (M.-K.S.); kmchoi@sookmyung.ac.kr (K.M.C.)

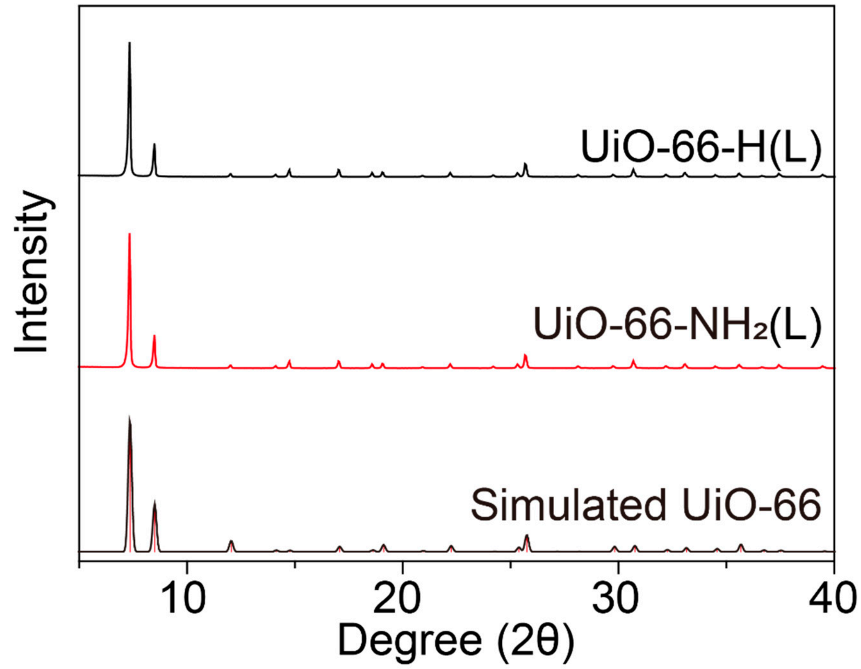

Figure S1. PXRD patterns of simulated UiO-66, UiO-66-H(L), and UiO-66-NH_2(L).

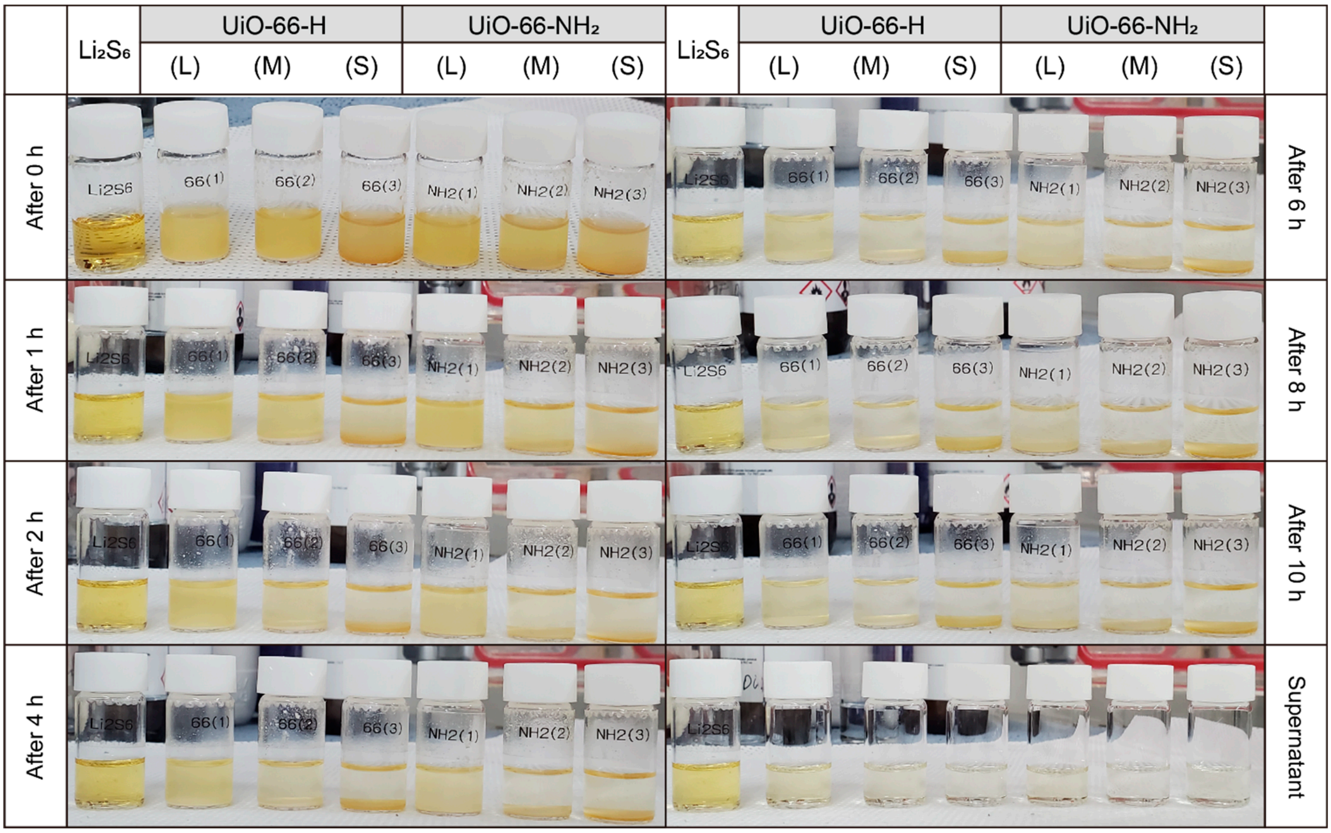

Figure S2. Digital images of 4 mM Li_2S_6 solution before and after soaking UiO-66s powders for 10 h for visual analysis of absorption.

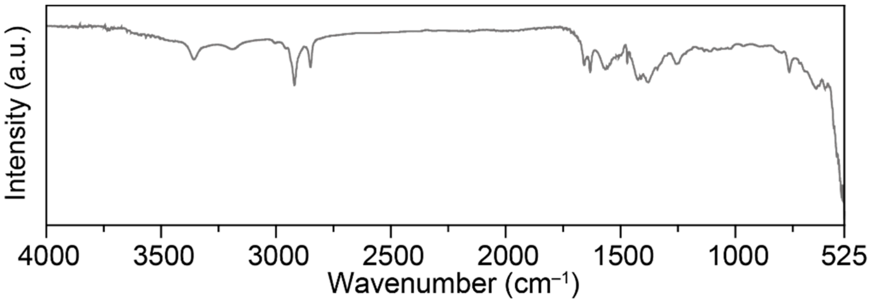

Figure S3. FT-IR spectra of Li_2S_6 solution after drying on a diamond ATR plate.

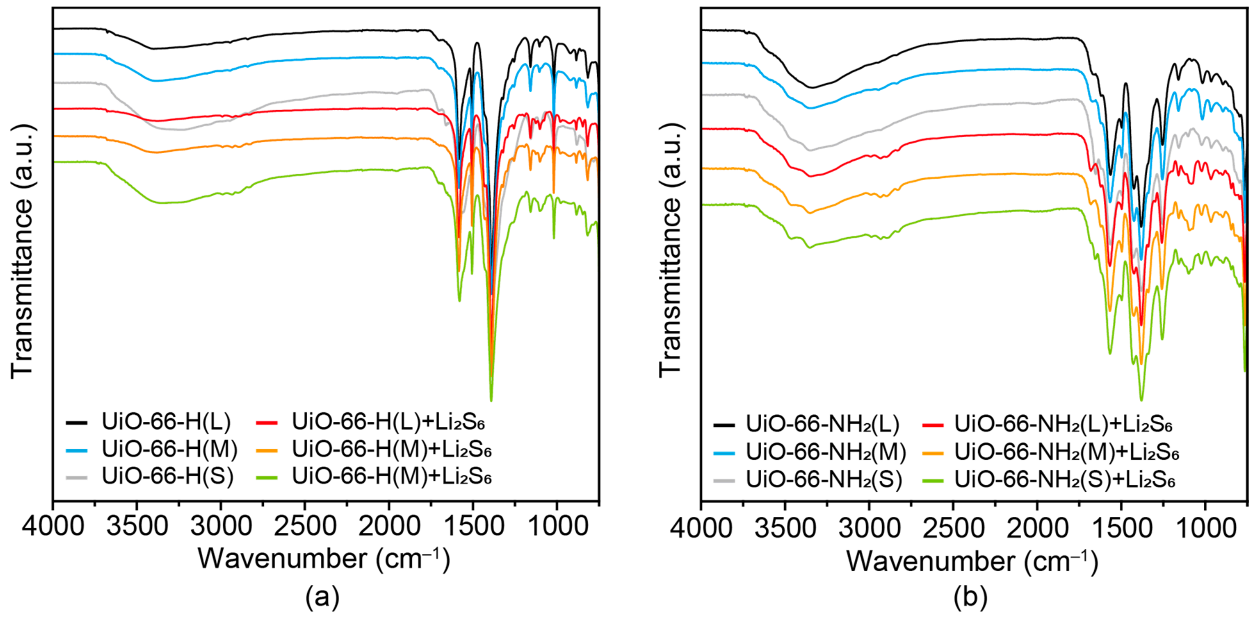

Figure S4. (**a**) FT-IR spectra of UiO-66-H(X) before and after absorbing polysulfide at 750–4,000 cm^{-1}. (**b**) FT-IR spectra of UiO-66-NH_2(X) before and after absorbing polysulfide at 750–4,000 cm^{-1}.

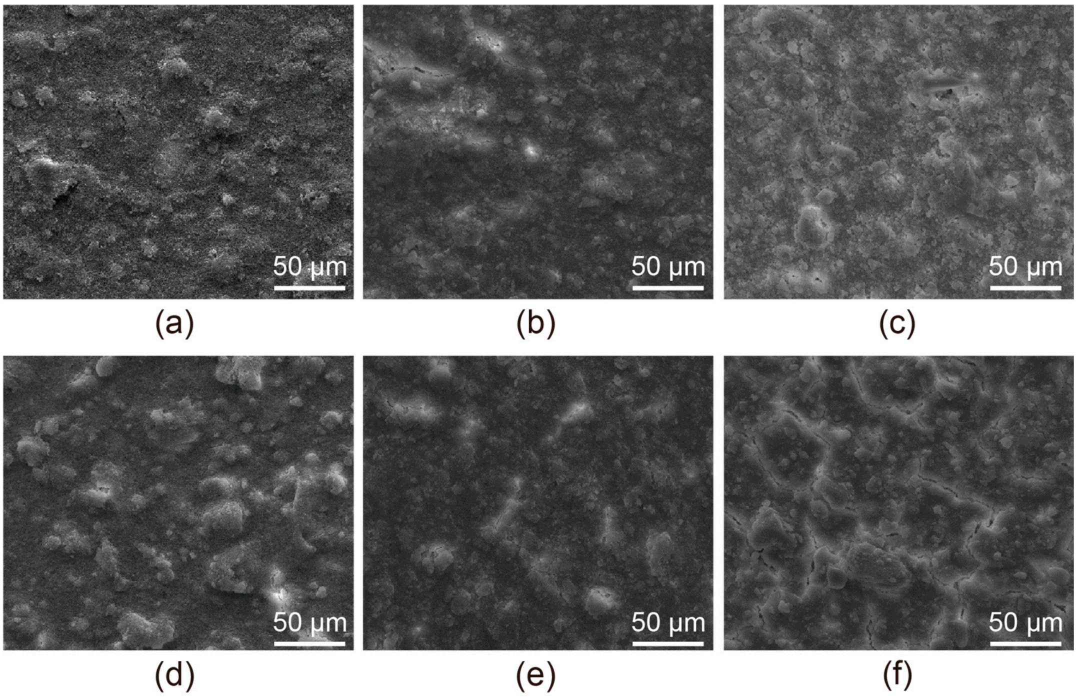

Figure S5. SEM images of the surface of MOF-coated separators made of (**a**) UiO-66-H(L), (**b**) UiO-66-H(M), (**c**) UiO-66-H(S), (**d**) UiO-66-NH_2(L), (**e**) UiO-66-NH_2(M), and (**f**) UiO-66-NH_2(S).

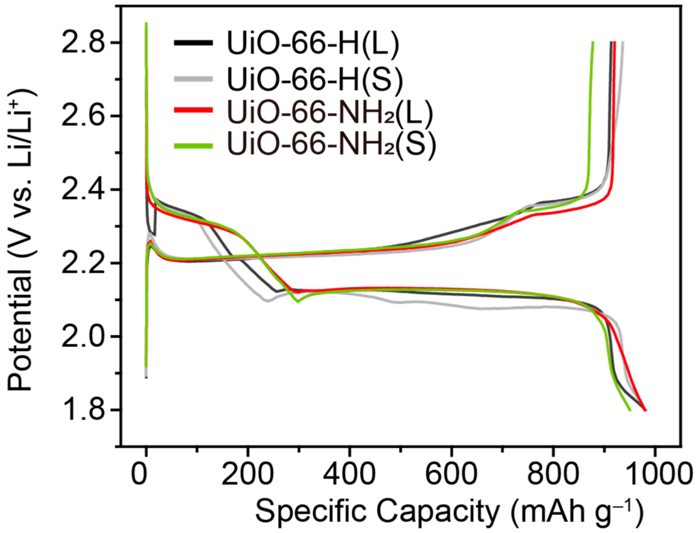

Figure S6. Voltage profiles of UiO-66-H(S),(L), and UiO-66-NH_2(S),(L) tested under 100 mA g^{-1}.

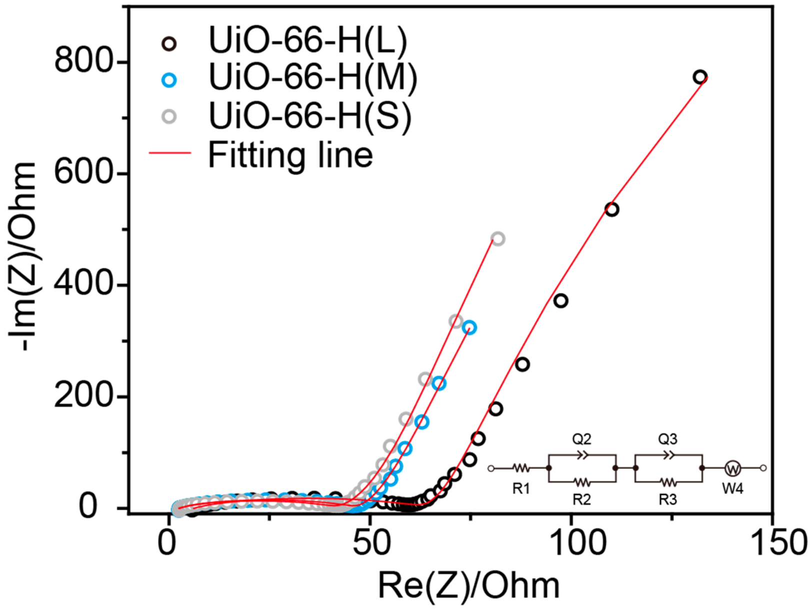

Figure S7. Nyquist plots of fresh Li–S cells with UiO-66-H(X) coated separators.

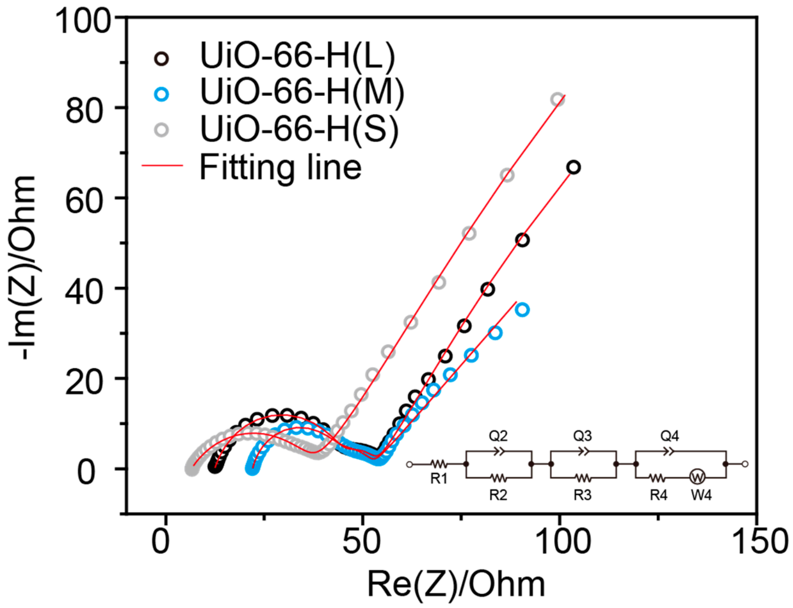

Figure S8. Nyquist plots of Li–S cells after 350 cycles with UiO-66-H(X)-coated separators.

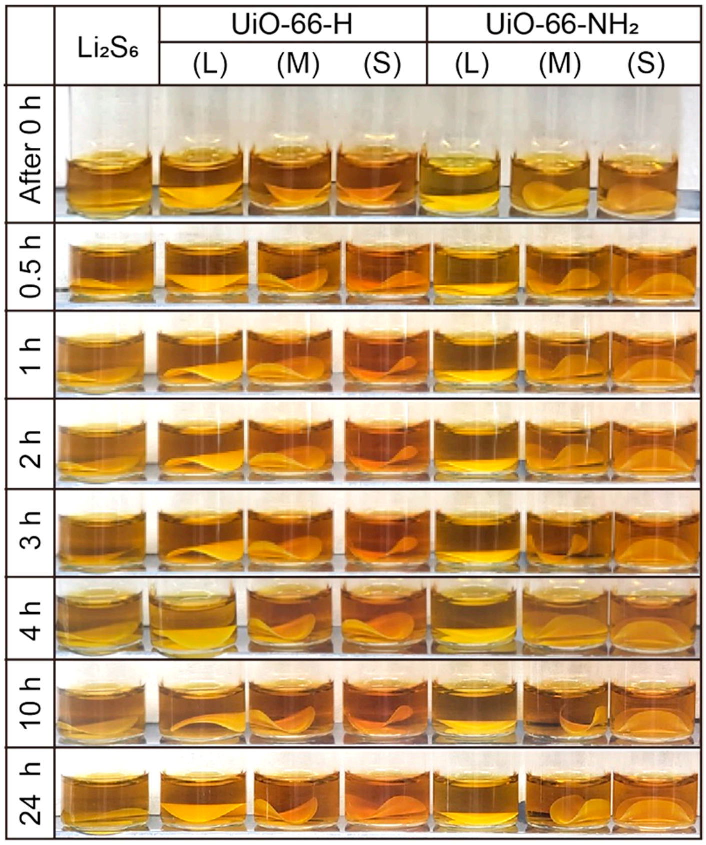

Figure S9. Digital images of Li_2S_6 solution before and after soaking MOF-coated separators for 24 h for visual analysis of absorption.

Table S1. Pore structure parameters of UiO-66s.

Sample	S_{BET} [1] (m^2 g^{-1})	V_P [1] (cm^3 g^{-1})	d_P [2] (nm)
UiO-66-H(L)	1292.8	0.5182	0.6
UiO-66-H(M)	1094.2	0.5804	0.6
UiO-66-H(S)	1091.7	1.0913	0.6
UiO-66-NH_2(L)	1040	0.4786	0.6
UiO-66-NH_2(M)	1024.9	0.7475	0.6
UiO-66-NH_2(S)	766.97	1.0259	0.6

[1] S_{BET} (BET surface area) and V_P (pore volume) were calculated by BET analysis.
[2] d_P (pore diameter) were calculated by MP plot.

Table S2. The comparison of electrochemical performance of reported UiO-66 coated separators in Li–S batteries.

MOFs	Separator substrate	Cathode materials	Mass loading of S (mg/cm^2)	Electrolyte (1/1, v/v)	C-rate	Cycle number	Ref.
UiO-66-H	Celgard 2500	S/Super P	1.5	1M LiTFSI in DOL/DME	0.2C (1C=1675 mA g^{-1})	720 mAh g^{-1} @500 cycles	[1]
UiO-66-H	Carbon cloth	S/CNT	2.1	1M LiTFSI in DOL/DME with 0.1M Li_2S_8	1C (1C=1675 mA g^{-1})	600 mAh g^{-1} after 600 cycles	[2]
UiO-66-S[1]	polyethylene membrane	S/rGO	1.7	1M LiTFSI in DOL/DME	0.2C (1C=1672 mA g^{-1})	437.6 mAh g^{-1} @200 cycles	[3]
UiO-66-SO_3Li	Free-standing	S/CMK-3	2.0	1M LiTFSI in DOL/DME with 2wt% $LiNO_3$	0.5C (1C=1675 mA g^{-1})	500 mAh g^{-1} @500 cycles	[4]
UiO-66-NH_2@SiO_2	Celgard 2320	S/ graphene	0.5	1M LiTFSI in DOL/TEGDME	0.1C (1C=1672 mA g^{-1})	600 mAh g^{-1} @100 cycles	[5]
UiO-66-NH_2@gra-phene	GF	S/CNT	1.4	1M LiTFSI in DOL/DME with 1wt% $LiNO_3$	1C (1C=1675 mA g^{-1})	500 mAh g^{-1} after 500 cycles	[6]
UiO-66-H (L); UiO-66-NH_2 (L)	Celgard 2400	S/CNT	0.6-1.0	1M LiTFSI in DOL/DME with 0.2M $LiNO_3$	0.15C (1C=1675 mA g^{-1})	271 mAh g^{-1} after 350 cycles; 420 mAh g^{-1} after 350 cycles	This work

[1] Sulfonic-acid functionalized UiO-66.

Table S3. EIS fitting parameters of fresh Li–S cells with UiO-66-H(X) coated separators.

	UiO-66-H(L)	UiO-66-H(M)	UiO-66-H(S)
R1 (Ω)	6.14	2.474	2.585
Q2 (F s^{a-1})	39.95e−6	26.71e−6	30.12e−6
a2	0.7162	0.7689	0.769
R2 (Ω)	56.3	43.21	38.06
Q3 (F s^{a-1})	4.492e−3	5.198e−3	5.172e−3
a3	0.9886	0.9906	1.0
R3 (Ω)	18167	22314	27568
Zw (Ω $s^{1/2}$)	14.76	16.28	21.35

Table S4. EIS fitting parameters of Li–S cells with UiO-66-H(X) coated separators after 350 cycles.

	UiO-66-H(L)	UiO-66-H(M)	UiO-66-H(S)
R1 (Ω)	12.43	21.97	6.521
Q2 (F s^{a-1})	12.46e−6	8.829e−6	0.2231e−3
A2	0.7838	0.8417	0.6001
R2 (Ω)	33.1	22.0	29.7
Q3 (F s^{a-1})	0.1794	11.18	0.1653
A3	0.7769	0.517 3e−12	0.8
R3 (Ω)	221.1	231.5	298.6
Q4 (F s^{a-1})	1.438e−3	0.5417e−3	0.04394
A4	0.8329	0.7862	0.458
R4 (Ω)	6.894	8.145	8.582
Zw (Ω $s^{1/2}$)	6.906	9.265	29.93

References

1. Fan, Y.; Niu, Z.; Zhang, F.; Zhang, R.; Zhao, Y.; Lu, G. Suppressing the Shuttle Effect in Lithium–Sulfur Batteries by a UiO-66-Modified Polypropylene Separator. *ACS omega* **2019**, *4*, 10328, https://doi.org/10.1021/acsomega.9b00884.
2. Zheng, S.; Zhao, X.; Liu, G.; Wu, F.; Li, J. A multifunctional UiO-66@carbon interlayer as an efficacious suppressor of polysulfide shuttling for lithium–sulfur batteries, *Nanotechnology*, **2021**, *32*, 365404, https://doi.org/10.1088/1361-6528/ac06f7.
3. Kim, S.H.; Yeon, J.S.; Kim, R.; Choi, K.M.; Park, H.S. A functional separator coated with sulfonated metal–organic framework/Nafion hybrids for Li-S batteries. *J. Mater. Chem. A*, **2018**, *6*, 24971, https://doi.org/10.1039/C8TA08843H..
4. Wang, Z.; Huang, W.; Hua, J.; Wang, Y.; Yi, H.; Zhao, W.; Zhao, Q.; Jia, H.; Fei, B.; Pan, F. An Anionic-MOF-Based Bifunctional Separator for Regulating Lithium Deposition and Suppressing Polysulfides Shuttle in Li–S Batteries. *Small Methods* **2020**, *4*, 2000082, https://doi.org/10.1002/smtd.202000082.
5. Suriyakumar, S.; Stephan, A.M.; Angulakshmi, N.; Hassan, M.H.; Alkordi, M.H. Metal–organic framework@SiO_2 as permselective separator for lithium–sulfur batteries. *J. Mater. Chem. A*, **2018**, *6*, 14623, https://doi.org/10.1039/C8TA02259C.
6. Guo, S.; Xiao, Y.; Wang, J.; Ouyang, Y.; Li, X.; Deng, H.; He, W.; Zeng, Q.; Zhang, W.; Zhang, Q. Ordered structure of interlayer constructed with metal-organic frameworks improves the performance of lithium-sulfur batteries. *Nano Research*, **2021**, https://doi.org/10.1007/s12274-021-3372-5.
